# Quality of Life After Parathyroidectomy in Chronic Kidney Disease–Related Hyperparathyroidism: A Systematic Review and Meta‐Analysis

**DOI:** 10.1002/wjs.70211

**Published:** 2025-12-19

**Authors:** Wellington Alves Filho, Marília D'Elboux Guimarães Brescia, Felipe Ferraz Magnabosco, Murilo Catafesta das Neves, Sergio Samir Arap, Rodrigo Oliveira Santos, Janaína de Almeida Mota Ramalho, Fabio Luiz de Menezes Montenegro, Marcio Ribeiro Studart da Fonseca

**Affiliations:** ^1^ Department of Head and Neck Surgery Hospital Universitário Walter Cantidio (HUWC/EBSERH) Faculty of Medicine of the Federal University of Ceara (UFC) Fortaleza Brazil; ^2^ Parathyroid Unit—LIM‐28, Head and Neck Surgery Laboratory, Division of Head and Neck Surgery, Department of Surgery Hospital das Clinicas (HCFMUSP) University of São Paulo School of Medicine (FMUSP) São Paulo Brazil; ^3^ Department of Otolaryngology and Head and Neck Surgery Federal University of Sao Paulo São Paulo Brazil; ^4^ Hospital Sirio Libanês São Paulo Brazil; ^5^ Department of Clinical Medicine Faculty of Medicine of the Federal University of Ceara (UFC) Fortaleza Brazil

**Keywords:** chronic kidney disease, hyperparathyroidism, meta‐analysis, parathyroidectomy, quality of life, secondary hyperparathyroidism

## Abstract

**Background:**

Secondary and tertiary hyperparathyroidism (SHPT and THPT) are frequent complications of chronic kidney disease and kidney transplantation, often impairing quality of life (QoL) through bone pain, fatigue, and pruritus. Parathyroidectomy is the definitive treatment for refractory cases, yet its impact on patient‐reported QoL outcomes remains uncertain.

**Methods:**

We conducted a systematic review and meta‐analysis in accordance with PRISMA guidelines (PROSPERO CRD42025108038). Nine studies (*n* = 675) with validated QoL assessments and ≥ 6 months of follow‐up were included. QoL was measured using SF‐36, KDQOL, and Pasieka’s parathyroid assessment of symptoms (PAS). Standardized mean differences (SMDs) were calculated, with analyses of physical (PCS) and mental (MCS) component summary scores. Meta‐regression evaluated preoperative parathyroid hormone (PTH), calcium, and phosphorus as predictors of QoL change.

**Results:**

Parathyroidectomy significantly improved global QoL (Hedges' *g* = 1.05; 95% CI: 0.42–1.69; *p* = 0.0011), PCS (SMD = 0.85; 95% CI: 0.32–1.37; *p* < 0.001), and MCS (SMD = 0.40; 95% CI: 0.11–0.69; *p* = 0.001). PAS scores also improved (SMD = −1.66; 95% CI: −2.72 to −0.60; *p* = 0.004). Preoperative PTH, calcium, and phosphorus were not associated with postoperative QoL gains (*p* = 0.71, 0.54, 0.47). Both subtotal and total parathyroidectomy provided comparable benefits (*p* = 0.76).

**Conclusion:**

Parathyroidectomy leads to meaningful QoL improvements in CKD‐related hyperparathyroidism, regardless of surgical technique. Baseline biochemical markers do not predict postoperative gains. Standardized, long‐term studies of patient‐reported outcomes are needed to guide surgical decision‐making.

## Graphical abstract

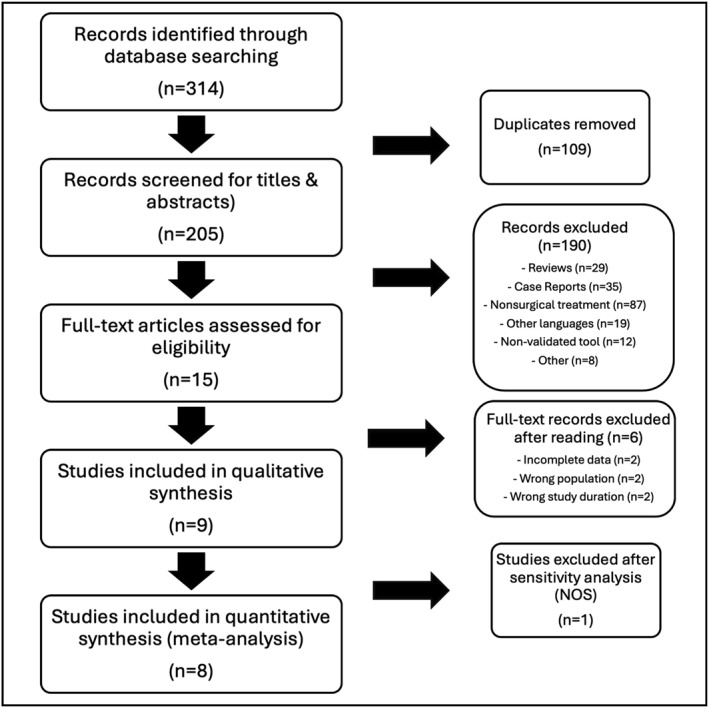

## 1 Introduction

Secondary and tertiary hyperparathyroidism (SHPT and THPT) are important complications of chronic kidney disease (CKD). In patients without access to renal transplantation or with persistent disease post‐renal transplantation, these conditions often become refractory to medical therapy [1]. Debilitating symptoms such as bone pain, fatigue, pruritus, and mood disturbances markedly impair quality of life (QoL) [2]. Chronically elevated parathyroid hormone (PTH) levels also contribute to vascular calcification, bone disease, and increased mortality, making SHPT and THPT key contributors to the systemic burden and poor outcomes seen in advanced CKD [3, 4].

When medical therapy fails to control SHPT and THPT, parathyroidectomy becomes the definitive treatment option. Surgical intervention is indicated in cases of refractory disease, severe biochemical abnormalities, or debilitating symptoms [5]. The primary goals of parathyroidectomy are to lower PTH levels and address the underlying metabolic disturbance, which is evidenced by abnormalities in biochemical markers including serum calcium and alkaline phosphatase [4]. In addition to improving bone metabolism and reducing cardiovascular risks, surgery has been shown to enhance QoL, addressing both physical limitations and mental health impairments commonly experienced by these patients [6, 7, 8, 9, 10].

Two main surgical strategies are used to treat SHPT and THPT: subtotal parathyroidectomy (STPT); and total parathyroidectomy (TPTX), often combined with autograft (TPTX + AG) [11, 12, 13, 14]. Both approaches have been shown to be effective in achieving biochemical control and improving QoL [6, 7, 8, 9, 15, 16, 17]. The choice between them typically depends on patients' anatomical characteristics, intraoperative findings, and surgeon experience, as both offer reliable outcomes in appropriately selected patients [18].

Although most studies report similar QoL outcomes between STPT and TPTX + AG, the existing evidence is limited by methodological heterogeneity. Differences in study design, follow‐up duration, and the use of various QoL instruments make direct comparisons difficult [10, 19, 20, 21, 22, 23, 24]. Furthermore, although QoL has been well documented in primary hyperparathyroidism [25], its evaluation in secondary and tertiary forms remains relatively neglected, despite these patients often experiencing greater symptom burden and systemic complications.

To date, no meta‐analysis has specifically focused on QoL outcomes after parathyroidectomy in SHPT and THPT. Notably, surgical treatment may offer superior QoL benefits in patients with advanced or refractory disease, where medical therapy alone often falls short [26]. This systematic review and meta‐analysis aim to fill a gap in the literature by evaluating the impact of parathyroidectomy on QoL in patients with SHPT and THPT, comparing outcomes between STPT and TPTX + AG, and exploring whether preoperative laboratory parameters predict changes in patient‐reported QoL.

## 2 Methods

### 2.1 Protocol and Registration

This systematic review and meta‐analysis was registered in the PROSPERO database (CRD42025108038) prior to data extraction. The protocol defined the review question, eligibility criteria, and planned methods in advance, ensuring methodological transparency and reducing the risk of bias. The review followed PRISMA guidelines and was conducted independently by two reviewers.

### 2.2 Search Strategy and Study Selection

A systematic literature search was performed across five electronic databases: MEDLINE, PubMed, Embase.com, LILACS, and the Cochrane Central Register of Controlled Trials (CENTRAL). The search strategy included a combination of controlled vocabulary and free‐text terms using the Boolean expression: (“secondary hyperparathyroidism” OR “tertiary hyperparathyroidism”) AND (“parathyroidectomy”) AND (“quality of life” OR “QoL”). Only articles published in English were included, with no restrictions on publication date. The last search was conducted on May 6, 2025. All records were imported into Rayyan for deduplication and screening. Two independent reviewers (W.A.F. and M.R.S.F.) conducted title/abstract and full‐text screening based on predefined eligibility criteria. Disagreements were resolved by consensus with a third reviewer.

### 2.3 Eligibility Criteria

Studies were eligible for inclusion if they met the following criteria: (1) involved adult patients (≥ 18 years) with a diagnosis of secondary or tertiary hyperparathyroidism; (2) evaluated the impact of STPT or TPTX + AG; (3) reported QoL outcomes assessed preoperative and/or postoperatively using validated instruments (e.g., SF‐36, KDQOL, PAS). Reports using nonvalidated or single‐item measures (e.g., unstructured symptom scores or VAS without validation) or lacking extractable pre/postoperative data were excluded; (4) had a minimum postoperative follow‐up of 6 months; and (5) employed a randomized controlled, cohort, or case–control study design. Studies were excluded if they did not clearly involve parathyroidectomy, included mixed surgical and nonsurgical cohorts without extractable surgical data, used nonvalidated QoL instruments, or did not provide preoperative or postoperative QoL data. When parathyroidectomy was confirmed but the study did not specify STPT vs. TPTX + AG, QoL data were included for pooled (overall) surgical effects and excluded from technique‐specific subgroup analyses. We also excluded case reports, small case series (< 10 patients), reviews, editorials, or conference abstracts. Only studies published in English were considered eligible.

### 2.4 Surgical Technique Definitions and Laboratory Parameters

Serum laboratory values were extracted and reported in standard international units: PTH in picograms per milliliter (pg/mL), calcium in milligrams per deciliter (mg/dL), and phosphorus in milligrams per deciliter (mg/dL).

Surgical strategies were defined as follows:

- TPTX + AG refers to the complete removal of all identifiable parathyroid tissue, followed by AG of parathyroid fragments into a heterotopic site (e.g., forearm or sternocleidomastoid muscle);
- STPT generally involves resection of three and a half parathyroid glands, leaving a small remnant of viable tissue in situ to preserve some parathyroid function. Although definitions of STPT varied slightly among included studies, this definition was used as the standard for data classification and analysis.

### 2.5 Outcomes

The primary outcome of interest was the change in QoL after STPT or TPTX + AG in patients with SHPT or THPT. QoL was assessed using only validated instruments, including the following:

- SF‐36 (Short Form‐36 Health Survey): a widely used generic tool measuring eight domains of health status. It provides two summary scores: the physical component summary (PCS), reflecting physical functioning, pain, and general health; and the mental component summary (MCS), assessing emotional well‐being, vitality, and social functioning.
- KDQOL (kidney disease quality of life instrument): a disease‐specific tool combining general health measures with kidney‐specific domains, such as symptoms, burden of kidney disease, and effects on daily life. Like the SF‐36, it includes PCS and MCS scores.
- PAS (parathyroid assessment of symptoms): a symptom‐specific tool evaluating the burden of parathyroid‐related complaints (e.g., bone pain, fatigue, pruritus) before and after surgery.

Secondary outcomes included the association between preoperative laboratory markers (e.g., PTH, calcium, phosphorus) and postoperative biochemical changes, as well as their potential predictive value for QoL improvement. To ensure that results reflected stable recovery rather than early postoperative fluctuations such as hungry bone syndrome, all included studies assessed QoL at a minimum of 6 months after surgery, with follow‐up periods extending up to 60 months. Where available, differences in QoL outcomes between surgical techniques (TPTX + AG vs. STPT) were explored, along with symptom‐specific improvements and multidimensional effects captured by PCS and MCS domains.

### 2.6 Data Extraction, Quality Assessment, and Risk of Bias

Data extraction was performed independently by one reviewer and verified by a second reviewer to ensure accuracy and completeness. Extracted information included study characteristics (e.g., author, year, country, design), patient demographics, type of hyperparathyroidism (secondary or tertiary), surgical approach (TPTX + AG or STPT), follow‐up duration, QoL assessment tools used, and preoperative and postoperative biochemical values (PTH, calcium, phosphorus). Outcomes related to changes in QoL and laboratory parameters were recorded, as well as subgroup data when available.

The methodological quality of included studies was assessed using the Newcastle–Ottawa Scale (NOS). Two reviewers independently rated each study, and disagreements were resolved by a third reviewer. NOS scores of 7–9 were considered high quality. Risk of publication bias was evaluated using funnel plot analysis and Egger's test when applicable.

### 2.7 Data Synthesis and Statistical Analysis

To enable comparison across different QoL instruments, standardized mean differences (SMDs; Hedges' g) were calculated for all continuous outcomes, following Cochrane guidelines. Because SF‐36, KDQOL, and PAS assess related aspects of well‐being using different scales, SMDs allowed pooling of comparable constructs of physical and mental health. Score directionality was harmonized by inverting PAS values so that higher scores uniformly indicated better QoL. The global QoL estimate represents standardized preoperative to postoperative differences across instruments. Follow‐up ranged from 6 to 60 months, and sensitivity analyses excluding shorter follow‐ups yielded consistent results. Meta‐analyses were performed using random‐effects models to account for heterogeneity between studies. Statistical heterogeneity was assessed using the I^2^ statistic, with values > 50% considered substantial. Subgroup analyses were conducted according to surgical strategy (TPTX + AG vs. STPT) and QoL domains (PCS, MCS, global/symptom‐specific), and meta‐regression explored demographic and biochemical predictors (age, sex, preoperative PTH). A *p* < 0.05 was considered statistically significant.

### 2.8 Certainty of Evidence

The certainty of evidence for each outcome was assessed using the GRADE (Grading of Recommendations, Assessment, Development, and Evaluations) framework. This approach considers five domains: risk of bias, inconsistency, indirectness, imprecision, and publication bias. For each main and additional outcome, the quality of evidence was rated as high, moderate, low, or very low. Randomized controlled trials initially contributed high‐certainty evidence, whereas observational studies started as low‐certainty and were upgraded or downgraded based on the presence or absence of concerns in each GRADE domain. Inconsistency was primarily judged based on heterogeneity statistics (e.g., I^2^), and imprecision was assessed by evaluating the width of confidence intervals and the total number of participants. Funnel plots and Egger's tests were used to inform judgments about publication bias.

## 3 Results

**FIGURE 1:**
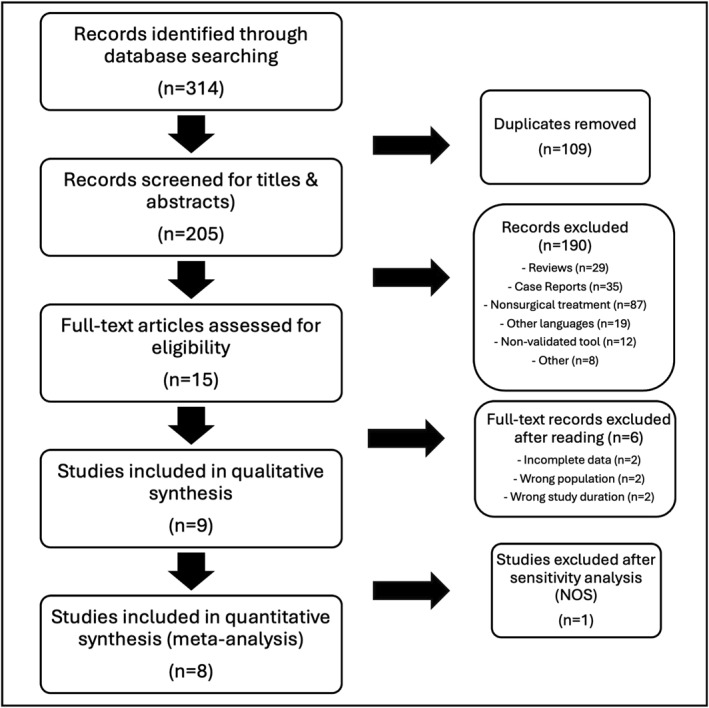
PRISMA flowchart of study selection.

A total of 314 records were identified through database searches. After removing duplicates and screening titles and abstracts, 15 studies were retrieved for full‐text review. Of these, 10 met the eligibility criteria and were included in the qualitative synthesis. After sensitivity analysis, one study was excluded, and the remaining 9 studies were included in the quantitative synthesis. All studies reported QoL outcomes using validated tools such as SF‐36, KDQOL, or PAS, with a minimum follow‐up of 6 months. Although most studies were observational, two were randomized controlled trials. Overall methodological quality was high in all studies (NOS 7–9). Meta‐regression showed no association between study quality and effect size (*p* = 0.59). Funnel plot inspection (Figure S1) and Egger's test revealed no evidence of publication bias (*p* = 0.84), and the certainty of evidence for QoL improvement was rated as moderate according to the GRADE approach (Table S1), based on the magnitude and consistency of the effect (Hedges' *g* = 1.98; 95% CI: 1.26–2.70). The study selection process is summarized in the PRISMA flowchart in Figure 1.

A total of 9 studies including 675 patients were analyzed, with 526 undergoing TPTX + AG, 66 undergoing STPT, and 83 with an unspecified technique. Mean patient age ranged from 46 to 56 years across studies, and the sex distribution was balanced (53.3% female overall). Follow‐up periods varied between 6 and 60 months. Preoperative PTH levels ranged widely depending on the population, with most studies reporting means between 1150 and 1800 pg/mL. Although two studies included only STPT patients and four included only TPTX + AG patients, the remaining studies reported outcomes from both surgical approaches. An overview of the included studies and patient characteristics is presented in Table 1 [6, 7, 8, 27, 28, 29, 30, 31, 32].

**TABLE 1:** Characteristics of included studies evaluating QoL after parathyroidectomy in CKD‐related hyperparathyroidism.

Study	Year	Country	Study design	Surgical approach	QoL tool used	*N*	Age (mean)	STPT/TPTX + AG	Sex (M/F)	Preop PTH (pg/mL)	Follow‐up (m)
Bratucu et al. [8]	2015	Romania	Prospective cohort	TPTX + AG	SF‐36; PAS	85	51	0/85	39/46	1379 (414–3345)	6
Cheng et al. [7]	2014	Taiwan	Prospective cohort	STPT; TPTX + AG	SF‐36; PAS	49	52	N/A	18/31	1416 ± 449	12
Filho et al. [6]	2018	Brazil	RCT	STPT; TPTX + AG	SF‐36	69	48	23/46	30/39	1478 ± 421	12
Jimeno‐Fraile et al. [27]	2021	Spain	Prospective cohort	STPT	SF‐36	23	55	23/0	16/7	1153 ± 667	6
Morandi et al. [28]	2024	Italy	Retrospective cohort	STPT; TPTX + AG	SF‐36; PAS	34	52	N/A	18/16	643 ± 327	24
Qiu et al. [29]	2023	China	Retrospective cohort	STPT; TPTX + AG	SF‐36	80	46.3	20/60	44/36	1239.4 (1621.8–2322.3)	12
See et al. [30]	2019	Singapore	Prospective cohort	TPTX + AG	PAS	91	54.9	0/91	37/54	211 ± 94.0	12
Wang et al. [31]	2022	China	Prospective cohort	TPTX + AG	KDQOL	212	46.4	0/212	96/116	1812 (1171.25–2640.73)	60
Wang et al. [32]	2023	Hong Kong	RCT	TPTX + AG	KDQOL	32	56	0/32	12/20	1643.5 ± 559	12

**FIGURE 2:**
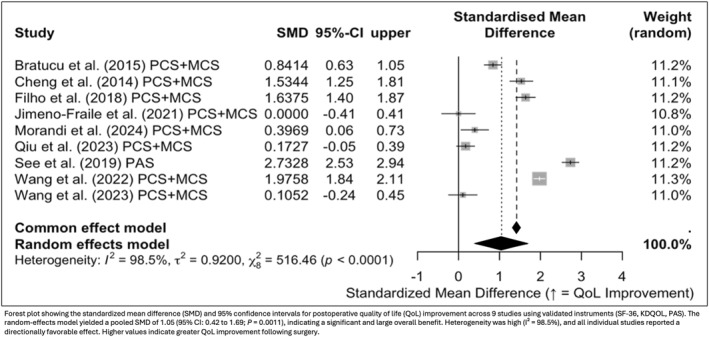
Overall improvement in quality of life after parathyroidectomy.

A random‐effects meta‐analysis demonstrated a significant overall improvement in QoL after surgery. The pooled standardized mean difference (SMD) was 1.05 (95% CI: 0.42 to 1.69; *p* = 0.0011), indicating a large positive effect (Figure 2). Substantial heterogeneity was observed (*I* ^2^ = 98.5%), likely due to variations in patient populations, assessment instruments, and surgical strategies. In a sensitivity analysis excluding two studies that included patients with THPT [28, 30], the pooled effect remained robust and highly significant (SMD = 1.68; 95% CI: 1.53 to 1.84; *p* < 0.0001), with negligible heterogeneity (*I* ^2^ ≈ 0%). Meta‐regression analyses showed that neither mean age (*p* = 0.825) nor sex (proportion of female patients; *p* = 0.691) significantly influenced QoL improvement, suggesting that these demographic factors did not explain between‐study variability. Despite heterogeneity in the broader dataset, all studies reported consistent directionality of effect, supporting the beneficial impact of parathyroidectomy on patient‐reported QoL.

**FIGURE 3:**
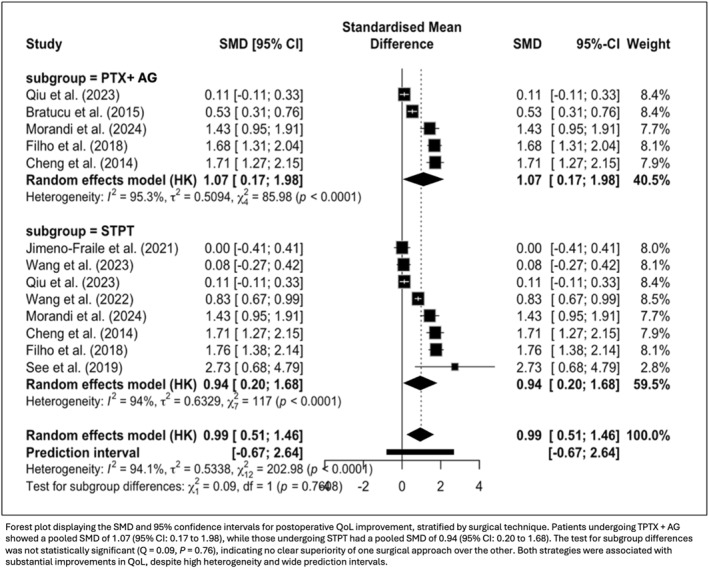
Subgroup analysis of postoperative quality of life improvement by surgical technique.

In a subgroup analysis of 13 study arms regarding surgical technique employed, both TPTX + AG and STPT were associated with significant improvements in postoperative QoL. The pooled SMD was 1.07 (95% CI: 0.17 to 1.98; *p* = 0.021) for TPTX + AG and 0.94 (95% CI: 0.20 to 1.68; *p* = 0.013) for STPT. The test for subgroup differences was not statistically significant (*Q* = 0.09; *p* = 0.76), indicating no clear advantage of one surgical technique over the other (Figure 3). Although heterogeneity remained considerable across studies, the consistent direction of effect supports the beneficial role of both surgical strategies in enhancing QoL.

**FIGURE 4:**
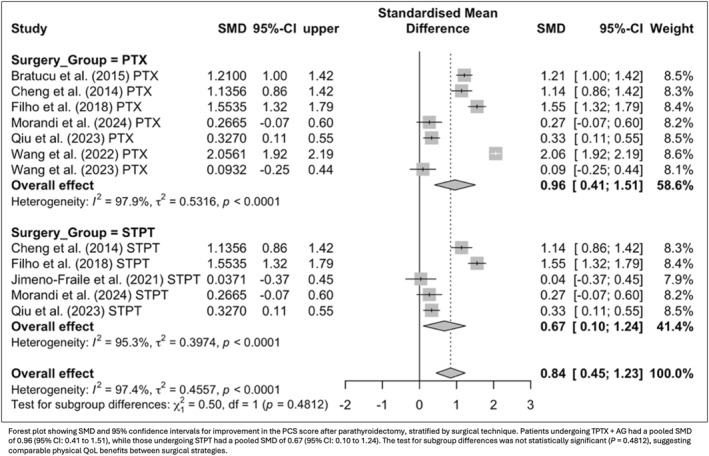
Subgroup analysis of physical component summary score (PCS) improvement by surgical technique.

**FIGURE 5:**
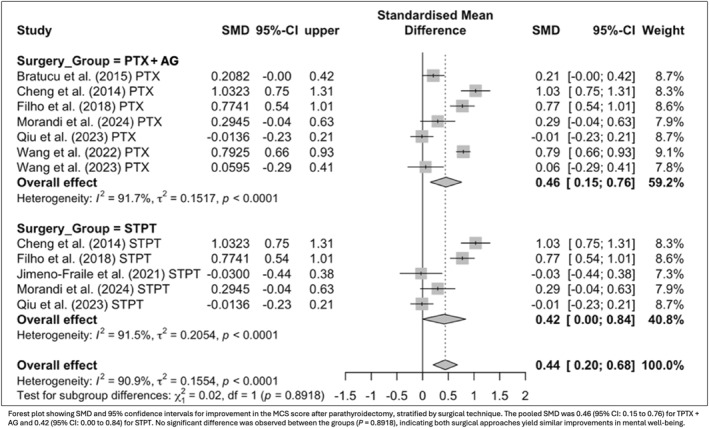
Subgroup analysis of mental component summary score (MCS) improvement by surgical technique.

To explore the multidimensional impact of surgery, postoperative QoL was evaluated across general health domains and symptom‐specific measures. Eight studies using the SF‐36 and KDQOL instruments demonstrated significant gains in both the PCS (SMD = 0.85; 95% CI: 0.32 to 1.37; *p* = 0.0015) and MCS (SMD = 0.40; 95% CI: 0.11 to 0.69; *p* = 0.007). In parallel, four studies assessing symptom burden through the PAS score reported a substantial reduction in complaints after surgery (SMD = −1.66; 95% CI: −2.72 to −0.60; *p* = 0.0021). When stratified by surgical technique, improvements in PCS were observed for both TPTX + AG (SMD = 0.96; 95% CI: 0.41 to 1.51; *p* = 0.0006) and STPT (SMD = 0.67; 95% CI: 0.10 to 1.24; *p* = 0.0212), with no significant difference between groups (*p* = 0.51; Figure 4). MCS improvements were also consistent across techniques (TPTX + AG: SMD = 0.46; 95% CI: 0.15 to 0.76; *p* = 0.0031; STPT: SMD = 0.42; 95% CI: 0.00 to 0.84; *p* = 0.049), likewise showing no significant difference between strategies (*p* = 0.89; Figure 5).

Preoperative laboratory values were strong predictors of their respective postoperative changes. Higher baseline PTH was linked to greater reductions post‐surgery (*β* = 1.97; 95% CI: 1.43 to 3.59; *p* < 0.001), and elevated calcium predicted a larger postoperative drop (*β* = 2.64; 95% CI: 1.42 to 3.86; *p* = 0.0018). Conversely, lower preoperative phosphorus levels were associated with greater increases after surgery (*β* = −1.30; 95% CI: −1.59 to −1.11; *p* = 0.018), reinforcing the utility of baseline biochemistry as a marker of surgical efficacy.

We evaluated the association between preoperative laboratory values and baseline QoL. PTH showed a moderate but nonsignificant negative correlation with PCS (*r* = −0.44; *p* = 0.27) and no correlation with MCS (*r* = −0.09; *p* = 0.83). Calcium was weakly and nonsignificantly correlated with both PCS (*r* = 0.28; *p* = 0.51) and MCS (*r* = 0.12; *p* = 0.78). In contrast, phosphorus demonstrated strong and significant correlations with both PCS (*r* = 0.84; *p* = 0.018) and MCS (*r* = 0.88; *p* = 0.0098); regression models showed that each 1 mg/dL increase in phosphorus corresponded to a 6.77‐point rise in PCS and a 2.28‐point rise in MCS. Among the biomarkers analyzed, phosphorus was the most robust predictor of baseline QoL.

However, these same biochemical parameters were not predictive of QoL improvement. No significant associations were observed between baseline PTH, calcium, or phosphorus and changes in global QoL, PCS, or MCS. Although baseline calcium was positively associated with MCS improvement in univariate models (*β* = 10.91; 95% CI: 2.65 to 21.16; *p* = 0.037), as was Δcalcium (*β* = 4.61; 95% CI: 1.89 to 10.87; *p* = 0.035), neither remained significant in multivariate analysis. Full meta‐regression findings are detailed in Tables 2 and 3.

**TABLE 2:** Correlation between preoperative laboratory markers and baseline PCS and MCS.

Laboratory predictor	Baseline PCS			Baseline MCS		
	r	95% CI	*p*	r	95% CI	*p*
PTH	−0.44	−0.87 to 0.38	0.27	−0.09	−0.75 to 0.65	0.83
Calcium	0.28	−0.63 to 0.82	0.51	0.12	−0.64 to 0.76	0.78
Phosphorus	0.84	0.24 to 0.98	0.018	0.88	0.36 to 0.98	0.0098

*Note:* This table summarizes the Pearson correlation coefficients (r), 95% confidence intervals (CI), and corresponding *p*‐values for the associations between preoperative parathyroid hormone (PTH), calcium, and phosphorus levels with preoperative physical (PCS) and mental (MCS) component summary scores of the SF‐36 quality of life instrument.

**TABLE 3:** Meta‐regression analysis of preoperative and postoperative biochemical markers as predictors of QoL improvement after parathyroidectomy.

Predictor	Univariate		Multivariate	
	Outcome	*p*‐value	Outcome	*p*‐value
Preop PTH	ΔQoL/PCS/MCS	0.71/0.89/0.35	PCS/MCS	0.73/0.96
Preop calcium	ΔQoL/PCS/MCS	0.54/0.42/0.037	PCS/MCS	0.51/0.40
Preop phosphorus	ΔQoL/PCS/MCS	0.47/0.54/0.86	PCS/MCS	0.41/0.62
ΔPTH	ΔQoL/PCS/MCS	0.86/0.40/0.63	PCS/MCS	0.84/0.63
ΔCalcium	ΔQoL/PCS/MCS	0.60/0.091/0.035	PCS/MCS	0.37/0.12
ΔPhosphorus	ΔQoL/PCS/MCS	0.60/0.17/0.27	PCS/MCS	0.32/0.50

*Note:* This table summarizes univariate and multivariate meta‐regression models evaluating the association between preoperative (PTH, calcium, phosphorus) and postoperative changes (ΔPTH, Δcalcium, Δphosphorus) in biochemical markers and improvements in QoL, assessed by global scores (ΔQoL), PCS, and MCS. *p*‐values are reported for each model. Although no marker showed consistent predictive value across outcomes, baseline calcium and Δcalcium were significantly associated with MCS improvement in univariate analysis (*p* = 0.037 and *p* = 0.035, respectively). However, no association remained significant in multivariate models.

## 4 Discussion

SHPT and THPT are common and disabling complications in patients with chronic kidney disease, including those on dialysis and post‐renal transplantation. Symptoms such as bone pain, fatigue, pruritus, sleep disturbances, and cognitive impairment contribute to a marked decline in QoL [33]. These issues often persist despite optimized medical therapy, particularly in advanced or refractory cases, where surgical intervention may play a key role in improving patient well‐being [2].

In this meta‐analysis, parathyroidectomy led to a significant and clinically meaningful improvement in overall QoL among patients with SHPT or THPT. Across nine studies using validated tools such as SF‐36, KDQOL, and PAS, the pooled effect size for global QoL improvement was large (Hedges' *g* = 1.05; 95% CI: 0.42–1.69). Both STPT and TPTX + AG were associated with significant benefits, with no statistically significant difference between techniques (*p* = 0.76). These findings are consistent with prior studies, including those by Filho et al. [6] and Cheng et al. [7], which reported comparable QoL gains regardless of surgical approach. Together, the results suggest that parathyroidectomy is effective in enhancing patient‐reported outcomes and that either technique may be selected based on clinical and institutional considerations without compromising benefit.

These benefits extended across multiple domains of QoL. Physical functioning improved significantly (*p* = 0.0015), and mental well‐being also showed meaningful gains (*p* = 0.007). Symptom burden, as measured by the PAS, was likewise reduced (*p* = 0.002), particularly in relation to bone pain, fatigue, and pruritus. Together, these multidimensional improvements highlight the impact of parathyroidectomy on both the physical and psychological aspects of life in patients with SHPT or THPT.

Preoperative laboratory values were closely associated with the extent of biochemical correction after surgery. Higher preoperative PTH and calcium levels predicted greater postoperative reductions (*p* < 0.001 and *p* = 0.0018, respectively), whereas lower phosphorus levels were linked to larger postoperative increases (*p* = 0.018). Among the laboratory markers, phosphorus was the only one significantly correlated with baseline QoL: For each 1 mg/dL increase in serum phosphorus, preoperative physical and mental component scores were 6.77 and 2.28 points higher, respectively. Although elevated serum phosphorus levels may indicate more severe hyperparathyroidism, this finding aligns with recent evidence showing that lower dietary phosphorus intake is associated with higher odds of symptoms, sleep disturbances, and reduced emotional well‐being, even after adjusting for confounders [34]. Notably, this relationship differs from the well‐established U‐shaped association between serum phosphorus levels and all‐cause mortality in patients with end‐stage kidney disease [35, 36]. In contrast, preoperative PTH demonstrated only a modest, nonsignificant inverse association with physical scores, consistent with some previous studies [6, 37]. Although these biochemical markers are valuable for assessing disease severity and predicting postoperative biochemical response, their relationship with perceived QoL appears inconsistent and is likely influenced by additional clinical and psychosocial factors.

Despite associations with baseline QoL and laboratory response, preoperative biochemical values did not predict postoperative QoL improvement. Meta‐regression showed no significant relationship between PTH, calcium, or phosphorus and gains in global QoL, PCS, or MCS. This contrasts with earlier reports suggesting that more severe abnormalities might yield greater recovery [2, 6]. A likely explanation is that subjective improvement depends not only on disease activity but also on comorbidities and psychosocial factors not captured by laboratory results [38]. In addition, variability in QoL instruments and follow‐up may attenuate associations in pooled data. Thus, although laboratory values guide surgical indication and monitoring, they do not predict the magnitude of QoL recovery.

This study has several limitations that should be acknowledged. First, the number of available studies assessing QoL after parathyroidectomy for SHPT and THPT remains limited. Despite the clinical relevance of this outcome, few trials have systematically measured QoL using validated instruments with appropriate follow‐up [6, 7]. The included studies were also heterogeneous in design, population, and follow‐up duration, which contributed to the substantial statistical heterogeneity observed in our pooled analyses. In addition, different QoL tools were employed, each with distinct domains and scoring methods. Some studies did not specify STPT vs. TPTX + AG, which precluded technique‐level attribution for a subset of patients; we, therefore, restricted those data to the overall surgical analyses. Some relevant studies had to be excluded due to limited data [9, 15], a follow‐up period shorter than 6 months [19, 20, 21], or the use of nonvalidated or subjective QoL measures [39], which may have introduced selection bias and limited generalizability. Moreover, patients with THPT may already experience better baseline QoL after renal transplantation, which could bias comparisons; thus, we performed a sensitivity analysis excluding studies with this population [28, 30]. The results of this analysis remained consistent with the overall findings, supporting the robustness of our conclusions.

Despite methodological challenges, this is the first meta‐analysis to specifically evaluate QoL outcomes after parathyroidectomy in patients with SHPT and THPT while also comparing the effects of different surgical techniques. Although another meta‐analysis has been registered on PROSPERO, it has not yet published results and does not include laboratory predictors or surgical subgroup analyses on its protocol [40]. A prior systematic review by van der Plas et al. [41] did compare surgical and pharmacological approaches, including cinacalcet, but did not perform a quantitative synthesis, limiting its ability to draw pooled conclusions. Although information on cinacalcet exposure was inconsistently reported among primary studies, its potential confounding influence should be acknowledged and explored in future research. In contrast, our study provides a comprehensive synthesis of QoL data, integrates clinical and biochemical predictors through meta‐regression, and directly compares STPT and TPTX + AG.

As newer minimally invasive treatments such as thermal ablation gain interest [42], it is essential to evaluate not only their biochemical efficacy but also their impact on patient‐reported outcomes. In contrast, parathyroidectomy remains a well‐established and cost‐effective intervention, providing significant QoL improvements across physical, mental, and symptom‐specific domains in SHPT and THPT [43]. Both subtotal and total procedures were effective, with no clear superiority between techniques. Although preoperative biochemical markers predicted laboratory changes and were variably linked to baseline QoL, they did not reliably forecast postoperative gains. Although this evidence does not define an optimal timing for surgery, the consistent QoL improvement observed suggests that patients with persistent impairment despite medical therapy may benefit from earlier referral for parathyroidectomy. These findings highlight the multifaceted nature of symptom perception and recovery in CKD‐related hyperparathyroidism and emphasize the need for future studies to incorporate standardized, patient‐centered QoL measures, as well as qualitative and mixed‐methods approaches, to capture patient narratives—such as fatigue, pruritus, sleep disturbance, and role function—that may not be fully represented by standardized instruments.

## Author Contributions

**Wellington Alves Filho:** conceptualization, methodology, writing – original draft, software, data curation, formal analysis. **Marília D'Elboux Guimarães Brescia:** writing – review and editing, formal analysis. **Felipe Ferraz Magnabosco:** writing – review and editing, formal analysis. **Murilo Catafesta das Neves:** writing – review and editing, formal analysis. **Sergio Samir Arap:** writing – review and editing, formal analysis. **Rodrigo Oliveira Santos:** writing – review and editing, formal analysis. **Janaína de Almeida Mota Ramalho:** writing – review and editing, formal analysis. **Fabio Luiz de Menezes Montenegro:** writing – review and editing, formal analysis, supervision. **Marcio Ribeiro Studart da Fonseca:** writing – original draft, data curation, formal analysis.

## Funding

The authors have nothing to report.

## Ethics Statement

The authors have nothing to report.

## Conflicts of Interest

The authors declare no conflicts of interest.

## Supporting information


**Figure S1**: Funnel plot for publication bias.

**Table S1**: Summary of evidence for primary and secondary outcomes.

## Data Availability

All data used in this systematic review and meta‐analysis were extracted from previously published studies, which are publicly available and cited within the manuscript. Extracted datasets and analysis files are available from the corresponding author upon reasonable request. All data needed to reproduce or recalculate pooled risk estimates are available and extractable from the published figures.
