# Supplementary figures and images for: Quality of Life After Parathyroidectomy in Chronic Kidney Disease–Related Hyperparathyroidism: A Systematic Review and Meta‐Analysis
Source: World J Surg. 2025 Dec 19;50(1):94–104. doi: 10.1002/wjs.70211 (PMC12831522; doi:10.1002/wjs.70211)

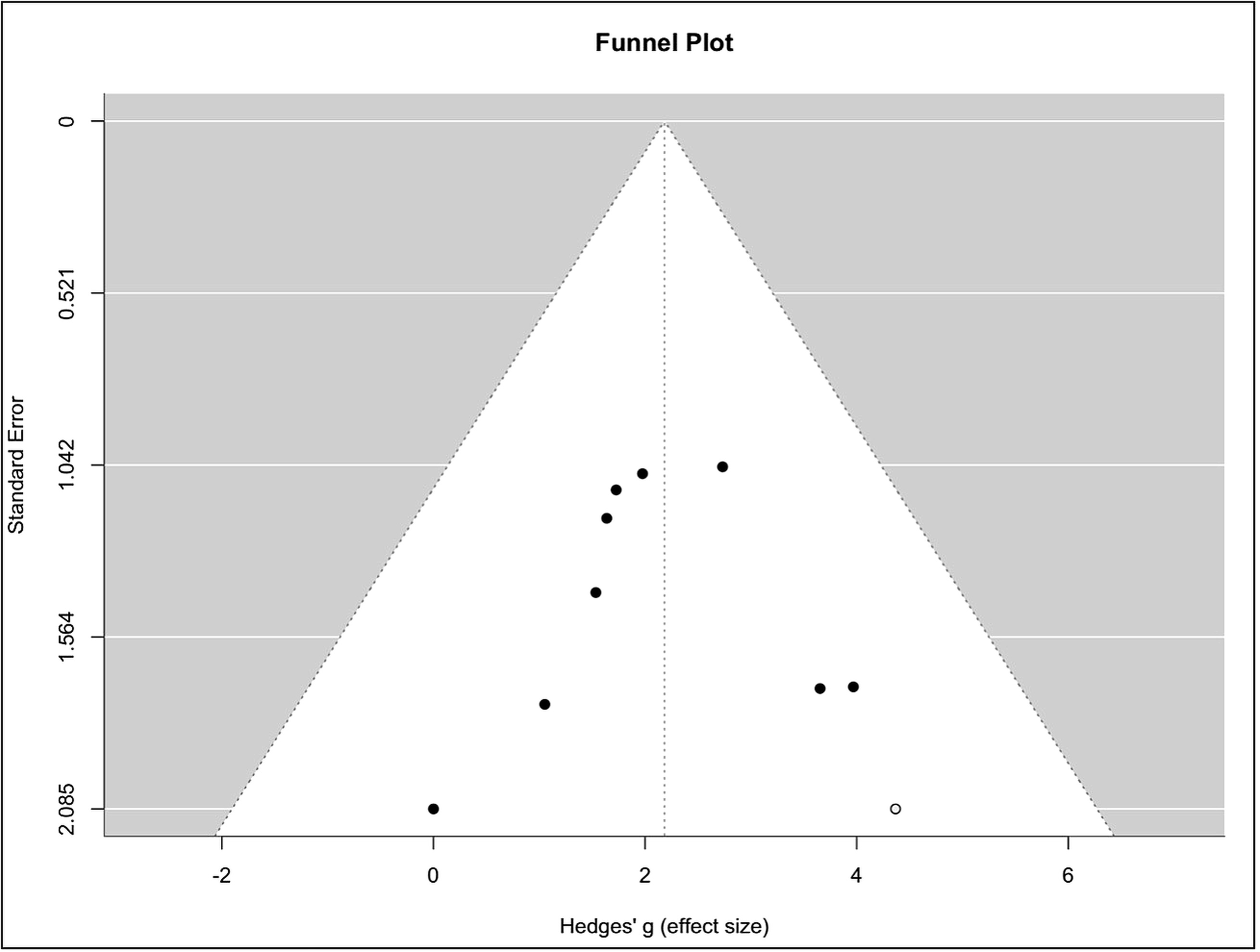

Supplement: Supplementary file 1 — Figure S1: Funnel plot for publication bias. [file WJS-50-94-s002.jpg]
