# Supplementary material for: Quality of Life After Parathyroidectomy in Chronic Kidney Disease–Related Hyperparathyroidism: A Systematic Review and Meta‐Analysis
Source: World J Surg. 2025 Dec 19;50(1):94–104. doi: 10.1002/wjs.70211 (PMC12831522; doi:10.1002/wjs.70211)
Supplement: Supplementary file 2 — Table S1: Summary of evidence for primary and secondary outcomes. [file WJS-50-94-s001.docx]

Supplementary Table S1. Summary of Evidence for Primary and Secondary Outcomes

| Outcome | No. of studies | Study design | Risk of bias | Publication bias | Effect size (95% CI) | Certainty |
| --- | --- | --- | --- | --- | --- | --- |
| QoL (global improvement) | 9 | Observational (Cohort/RCT) | Not serious | Undetected | Hedges' g = 1.05 (0.42 to 1.69) | MODERATE ⨁⨁⨁◯ |
| PCS | 8 | Observational (Cohort/RCT) | Not serious | Undetected | SMD = 0.85  (0.32 – 1.37) | MODERATE ⨁⨁⨁◯ |
| MCS | 8 | Observational (Cohort/RCT) | Not serious | Undetected | SMD = 0.40  (0.11 – 0.69) | MODERATE ⨁⨁⨁◯ |
| PAS | 4 | Observational (Cohort/RCT) | Not serious | Undetected | SMD = -1.66  (-2.72 – -0.60) | MODERATE ⨁⨁⨁◯ |

RCT: randomized controlled trial
